# The Type of Lateral Hinge Fracture in Medial Open-Wedge High Tibial Osteotomy Determines Its Stability: A Biomechanical Study

**DOI:** 10.1177/03635465251332593

**Published:** 2025-04-28

**Authors:** Christian Peez, Alexander Milstrey, Ivan Zderic, Adrian Deichsel, R. Geoff Richards, Boyko Gueorguiev, Christoph Kittl, Michael J. Raschke, Elmar Herbst

**Affiliations:** † AO Research Institute Davos, Davos, Switzerland; ‡ Department of Trauma, Hand and Reconstructive Surgery, University Hospital Münster, Münster, Germany; Investigation performed at AO Research Institute Davos, Davos, Switzerland

**Keywords:** medial open-wedge high tibial osteotomy, lateral hinge fractures, Takeuchi classification, torsional instability, biomechanics, motion tracking

## Abstract

**Background:**

Lateral hinge fractures (LHFs) are considered risk factors for delayed union or nonunion after medial open-wedge high tibial osteotomies (MOWHTOs). However, there is limited evidence on the extent to which the morphology of the hinge fracture influences the stability of a MOWHTO.

**Purpose/Hypothesis:**

The purpose of this study was to validate the Takeuchi classification under axial and torsional loading to identify the LHF types requiring surgical treatment. It was hypothesized that (1) LHFs would reduce construct stiffness and increase interfragmentary instability across the osteotomy gap, and (2) shear displacement associated with impaired bone healing of >2 mm would be observed in Takeuchi type 2 and 3 fractures.

**Study Design:**

Descriptive laboratory study.

**Methods:**

A total of 24 fresh-frozen human cadaveric proximal tibiae underwent MOWHTO fixed with a locking compression plate. The specimens were assigned to 3 different groups so that the mean bone mineral density values were similar between the groups. Each group simulated a different type of LHF according to the Takeuchi classification: (1) type 1 fracture, extension along the osteotomy plane; (2) type 2 fracture, extension distal to the proximal tibiofibular joint; and (3) type 3 fracture, proximal extension into the lateral tibial plateau. Each specimen was subjected to 10 quasi-static cycles of axial compression up to 720 N, followed by internal and external torsional loading up to 10 N·m, while the interfragmentary movements were captured with a motion tracking system.

**Results:**

Compared with a MOWHTO with a preserved lateral hinge, Takeuchi type 2 and 3 fractures significantly increased shear displacement and hinge rotation by 2.2 mm and 2.3°, respectively, resulting in at least 80% reduction in torsional stiffness (*P* < .001). In contrast, Takeuchi type 1 fractures did not significantly alter the torsional stability of a MOWHTO. Takeuchi type 2 and 3 fractures significantly increased axial displacement at the hinge site by 0.2 mm (*P* < .01) compared with an intact hinge MOWHTO, while axial displacement of the medial osteotomy gap remained unchanged. All Takeuchi types significantly reduced axial construct stiffness by at least 28% (*P* < .01).

**Conclusion:**

From a biomechanical perspective, Takeuchi type 1 LHFs did not affect the torsional stability of MOWHTO, whereas Takeuchi type 2 and 3 fractures resulted in significantly reduced torsional stiffness, increased shear displacement, and hinge rotation across the osteotomy gap. All Takeuchi fracture types resulted in reduced axial construct stiffness, while axial displacement was not significantly affected by the type of hinge fracture.

**Clinical Relevance:**

The observed shear displacement of >2 mm for Takeuchi type 2 and 3 fractures may be indicative of impaired bone healing and may therefore qualify these fractures for hinge fixation to potentially reduce the risk of delayed union and nonunion.

Lateral hinge fractures (LHFs) are a commonly observed complication in medial open-wedge high tibial osteotomy (MOWHTO), resulting in an increased risk of malunion and nonunion.^4,17,20,28,30,32^ These fractures at the hinge of the osteotomy site may occur with an incidence of up to 50% and present with different fracture types.^10,15-17,22,32^ In fact, Takeuchi fracture types 2 and 3 are considered unstable and are predisposed to impaired bone healing of the osteotomy gap, resulting in fatigue hardware failure, loss of correction, chronic postoperative pain, and thus poorer functional outcomes.^4,7,14,22,26,28,32^

Recent biomechanical studies have shown that LHFs significantly reduce the stability of the MOWHTO. In this context, the impaired bone healing has especially been attributed to the reduced axial and torsional stability of the bone-implant construct as well as the increased micromovements across the osteotomy gap.^3,7,13^ As a result, several strategies have been proposed to prevent these fractures, such as the use of prophylactic hinge wires or screws.^9,12^ However, hinge fractures remain a clinical problem, leading to poor functional and patient-reported outcomes.^9,21^ In these cases, restoring the primary stability of the osteotomy by an additional fixation of the fractured hinge represents a potential key to reduce the LHF-dependent bone healing failure.^7^ However, there is no consensus in the literature as to which fracture types are considered unstable, and therefore no fracture-specific treatment strategies are currently available.^3,7,13^

The aim of this study was to validate the Takeuchi classification under torsional and axial loading to define the fracture types requiring surgical treatment. It was hypothesized that (1) hinge fractures would reduce construct stiffness and increase interfragmentary instability across the osteotomy gap, and (2) shear displacement associated with impaired bone healing of >2 mm would be observed in Takeuchi type 2 and 3 fractures.

## Methods

A total of 24 fresh-frozen (–20°C) nonpaired human cadaveric knees from 6 female and 16 male donors aged 72.7 ± 7.1 years (mean value ± standard deviation (SD); range, 54-82 years) were obtained from an international tissue bank (Science Care). All donors gave their informed consent within the donation of anatomical gift statement during their lifetime.

The proximal tibiae of all knees were assessed for bone mineral density (BMD) within the trabecular region of the proximal tibial metaphysis using computed tomography (Revolution EVO; GE HealthCare). A BMD callibration phantom (BDC-6; QRM GmbH) was subsequently analyzed using image processing software (Amira Version 6.0; Thermo Fisher Scientific) with segmentation between 150 and 450 mgHA/cm^3^.

The knees were assigned to 3 groups consisting of 8 specimens each, with each group simulating a different type of LHF according to the Takeuchi classification: (1) Takeuchi type 1 fracture, (2) Takeuchi type 2 fracture, and (3) Takeuchi type 3 fracture.^32^ Block randomization was used to allocate the specimens to the different testing conditions, such that the BMD values were homogeneously distributed among the 3 groups. Allocation was based on BMD to ensure a high degree of standardization and thus comparability between the testing conditions.^11^

### Specimen Preparation

Before preparation and biomechanical testing, the knees were thawed at room temperature for 24 hours. After cutting the tibia and fibula 250 mm distal to the knee joint line, the knees were disarticulated to harvest the tibia and fibula. During dissection, special care was taken to protect the interosseous membrane and the joint capsule of the proximal tibiofibular joint. The fibula was secured to the tibia in its anatomic position by a tricortical 3.5-mm position screw. The screw was placed as distally as possible to mimic the syndesmosis and avoid free floating of the fibula. Specimens were wrapped in phosphate-buffered saline-soaked tissue paper to prevent tissue dehydration.

**Figure 1.**
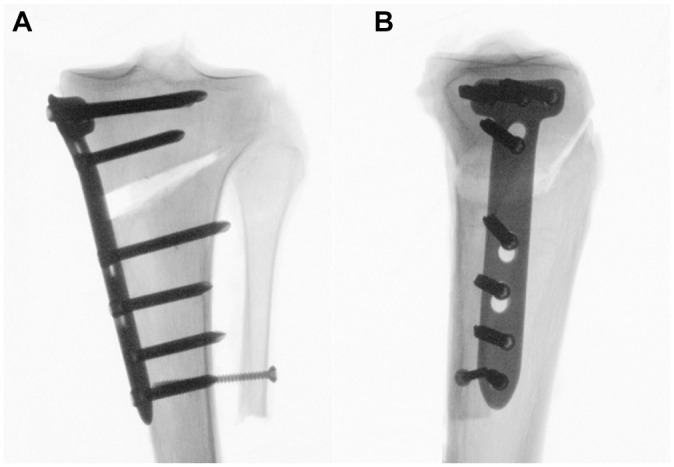
(A) Anteroposterior and (B) lateral radiographs of a left proximal tibia after a biplanar medial open-wedge high tibial osteotomy fixed with a locking compression plate. Particular care was taken to preserve an intact lateral hinge.

In each specimen, a biplanar MOWHTO was performed using the technique described by Palmer et al.^24^ The osteotomy plane was marked with 2 parallel 2.4-mm Kirschner (K-) wires with the tips placed lateral to the medial margin of the proximal tibiofemoral joint, marking the hinge position in the safe zone.^21^ For the biplanar cut, an ascending anterior osteotomy was performed behind the tibial tuberosity at an angle of 100° to the planned correction level, followed by an axial osteotomy along the K-wires using an oscillating saw. Consistent with previous studies, the osteotomy gap was opened to a height of 10 mm, while preserving an intact lateral hinge of 10 mm in width.^3^ Then, the MOWHTO was fixed using a medial 4.5/5.0-mm locking compression plate (LCP) system (TomoFix medial proximal tibia; Johnson & Johnson MedTech). Four unicortical locking screws were placed in the metaphyseal segment, while the diaphyseal segment was fixed with 4 bicortical locking screws (Figure 1).

On completion of the surgical procedures, the distal 6 cm of the tibial shafts was embedded in a polymethylmethacrylate (PMMA) (Suter Kunststoffe AG) socket. Then, the tibial plateau was embedded in a PMMA socket, with the lateral 50% of the lateral tibial plateau and the proximal tibiofibular joint being free of PMMA. For this purpose, the entire tibial plateau was first cemented with PMMA. After curing, the PMMA was carefully removed from the lateral half of the lateral tibial plateau and the proximal tibiofibular joint using a saw and chisel. Care was taken to avoid damaging the metaphyseal bone of the proximal tibia or the articular capsule of the proximal tibiofibular joint. Finally, retroreflective marker sets were attached to the tibial shaft, the lateral hinge site, and the medial proximal tibial metaphysis for motion tracking.

### Biomechanical Testing

**Figure 2.**
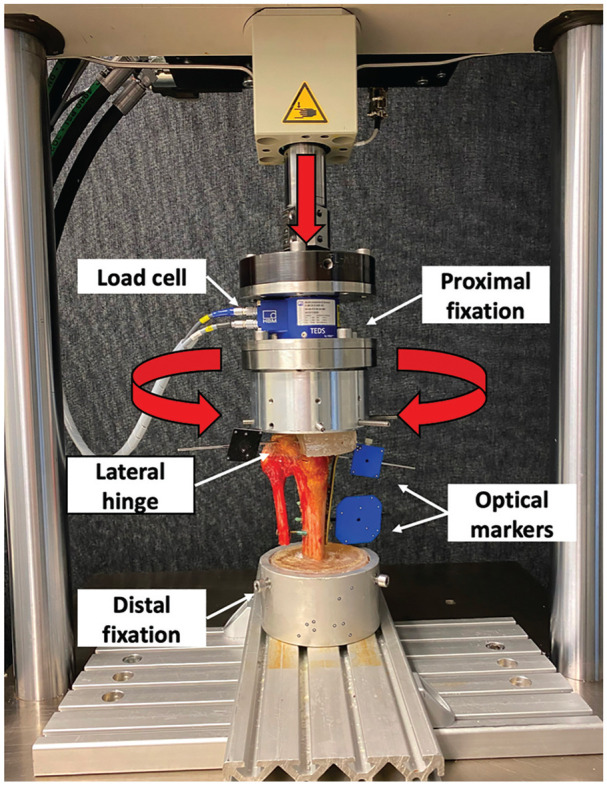
Setup with a specimen mounted for biomechanical testing. The vertical arrow denotes the axial loading direction, and the semicircular arrows denote bidirectional torsional loading.

Biomechanical testing was performed using a servohydraulic materials testing machine (Bionix 858.20; MTS Systems Corp) equipped with a 5-kN load cell (MCS; HBM) that allows position and force control with 0.05% accuracy. Each specimen was tested in an upright standing position, with the distal tibial PMMA socket rigidly mounted to the materials testing machine base plate. The proximal PMMA socket was mounted to the test actuator via a custom-made fixation device. As the lateral 50% of the lateral tibial plateau and the fibula including the proximal fibular joint were free of PMMA, axial compression and torsional loading were selectively applied to the metaphyseal segment of the MOWHTO, allowing homogeneous load transfer without restricting the mobility of the lateral hinge and the proximal tibiofibular joint (Figure 2).

**Figure 3.**
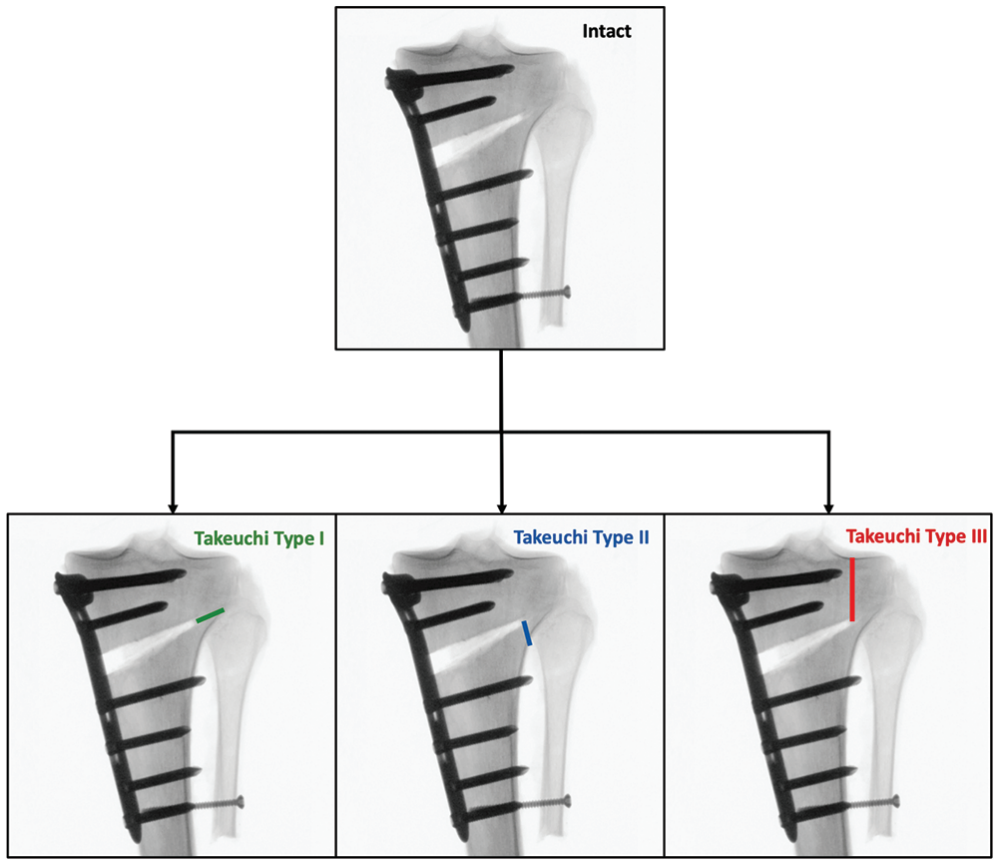
Schematic illustration denoting the workflow. After testing each specimen with an intact lateral hinge, the lateral hinge site was osteotomized according to the specimen’s group affiliation to simulate different Takeuchi types of lateral hinge fractures.^32^ The Takeuchi types are as follows: type 1 fracture, extension along the osteotomy plane into the proximal tibiofibular joint; type 2 fracture, extension distal to the proximal tibiofibular joint into the lateral cortex; and type 3 fracture, extension into the lateral tibial plateau proximal to the proximal tibiofibular joint.

Starting with a preserved hinge (intact), each specimen was axially loaded with 10 cycles of a nondestructive quasi-static ramp (720 N, 36 N/s), followed by torsional loading with 10 cycles of a nondestructive quasi-static ramp (10 N·m, 1 N·m/s) in internal and external rotation as previously described.^25^ Once the measurements were completed for the intact test condition, the lateral hinge site was osteotomized with an oscillating saw according to the specimen’s group affiliation to simulate different Takeuchi types of LHFs.^32^ While leaving the joint capsule of the proximal tibiofibular joint intact, the lateral hinge was osteotomized along the osteotomy plane, with the osteotomy extending laterally within the tibiofibular joint to simulate a Takeuchi type 1 fracture. To preserve the joint capsule of the proximal tibiofibular joint, the osteotomy was carefully completed with a chisel. A Takeuchi type 2 fracture was created by osteotomizing the lateral cortex of the proximal tibia distal to the proximal tibiofibular joint, whereas a Takeuchi type 3 fracture was simulated by an osteotomy of the lateral hinge extending proximally into the lateral tibial plateau (Figure 3). Then, the biomechanical testing and measurements were repeated for the different fracture types.

### Data Acquisition and Evaluation

Machine data in terms of axial load, axial displacement, torsional torque, and torsional angle were continuously acquired from the system controllers of the materials testing machine at 128 Hz throughout each test. Based on these data, force-displacement and torque-angle curves from the axial compression and the torsional tests were generated to calculate the construct axial and torsional stiffness, respectively, bothe defined as the slope of the initial quasi-static ramp within its linear range.

In addition, a stereographic optical motion tracking system using contactless full-field deformation technology (Aramis SRX; Carl Zeiss GOM Metrology GmbH) operating at a resolution of 12 megapixels and a maximum acceptance error of 0.004 mm continuously captured the coordinates of the attached markers in all 6 degrees of freedom at 20 Hz.^27^ Based on this, interfragmentary movements were evaluated over the 10 test cycles for each loading condition. Specifically, hinge fracture site displacement along the tibial shaft axis (defined as axial displacement) and perpendicular to it in the anteroposterior direction (defined as shear displacement) was captured. In addition, axial displacement of the medial osteotomy gap was captured. Interfragmentary rotation around the longitudinal axis of the tibial shaft (defined as hinge rotation) was also evaluated.

### Statistical Analysis

Statistical analysis was performed using commercial software (Prism Version 9; GraphPad Software). Descriptive data are presented as mean value and standard deviation. The normality of data distribution within each fixation technique was tested and proved using the Shapiro-Wilk test. Significant differences between the groups regarding BMD, axial and torsional construct stiffness, axial displacement, shear displacement, and hinge rotation were determined using 1-way analysis of variance followed by post hoc Tukey testing for multiple comparison. The overall level of significance was set at a *P* value <.05.

An a priori power analysis was performed using G*Power 2 software (University Düsseldorf, Düsseldorf, Germany).^5^ Based on the mean values and standard deviations of a previous biomechanical study evaluating hinge fracture displacement after distal femoral osteotomies, it was assumed that a sample size of 6 would allow the identification of changes in displacement of 0.3 mm with a standard deviation of 0.2 mm (effect size/Cohen *d* = 1.5) with 95% power, at the significance level of *P* < .05.^25^

## Results

The BMD was 141.3 ± 40.9 mgHA/cm^3^ for Takeuchi type 1, 148.3 ± 42.9 mgHA/cm^3^ for Takeuchi type 2, and 149.2 ± 41.8 mgHA/cm^3^ for Takeuchi type 3 fractures, demonstrating a homogeneous distribution among the groups.

### Axial Loading

**Figure 4.**
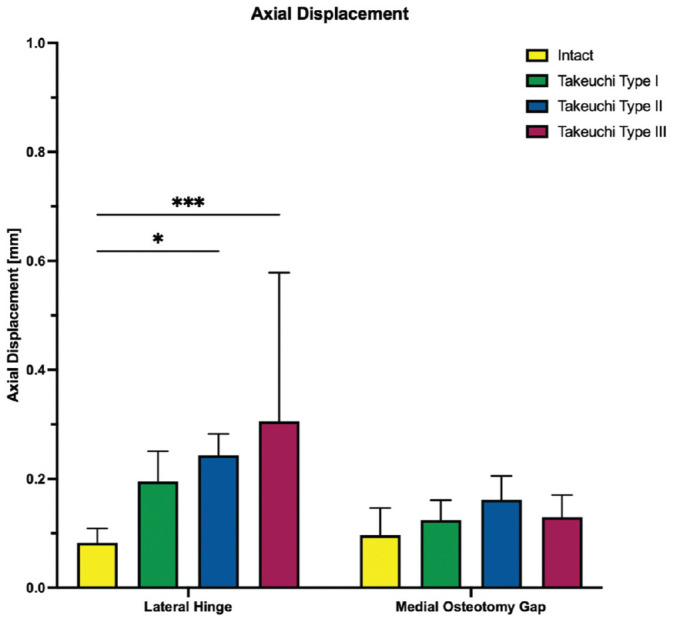
The axial displacement of the lateral hinge and medial osteotomy gap is shown for each testing condition separately. Values are given as mean ± SD. *P < .01; ***P < .001.

**Figure 5.**
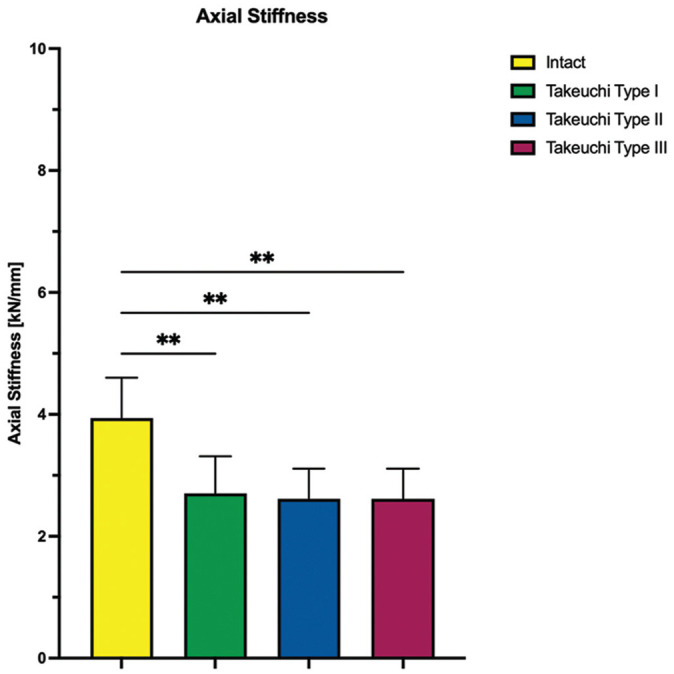
The axial stiffness is shown for each testing condition separately. Values are given as mean ± SD. **P < .01.

Takeuchi type 2 and 3 fractures significantly increased axial displacement at the lateral hinge site by at least 0.2 ± 0.1 mm compared with an MOWHTO with an intact lateral hinge (type 2: *P* < .01; type 3: *P* < .001), whereas Takeuchi type 1 fractures did not significantly affect axial displacement (*P* = .088). In addition, none of the LHF subtypes significantly increased axial displacement at the medial osteotomy gap (*P* > .467) (Figure 4). Conversely, all Takeuchi types of LHFs resulted in a significant reduction in axial construct stiffness of at least 28% compared with intact lateral hinges (type 1: *P* < .01; types 2 and 3: *P* < .01) (Figure 5).

### Torsional Loading

**Figure 6.**
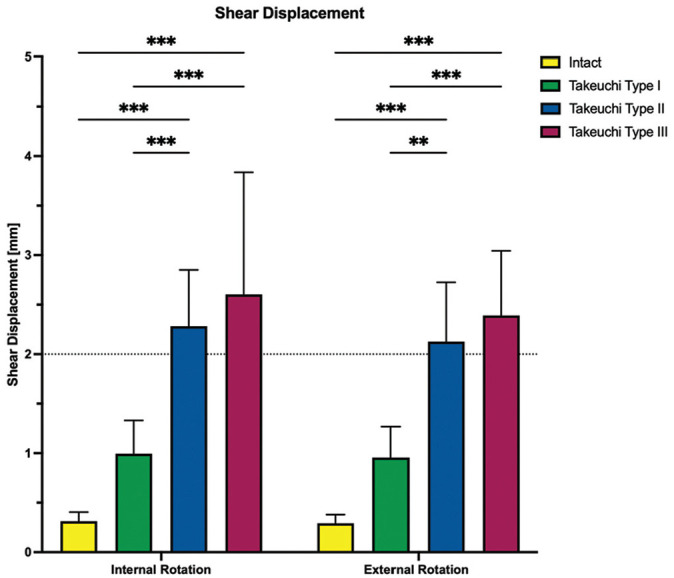
The shear displacement in internal and external rotation is shown for each testing condition separately. Values are given as mean ± SD. **P < .01; ***P < .001.

**Figure 7.**
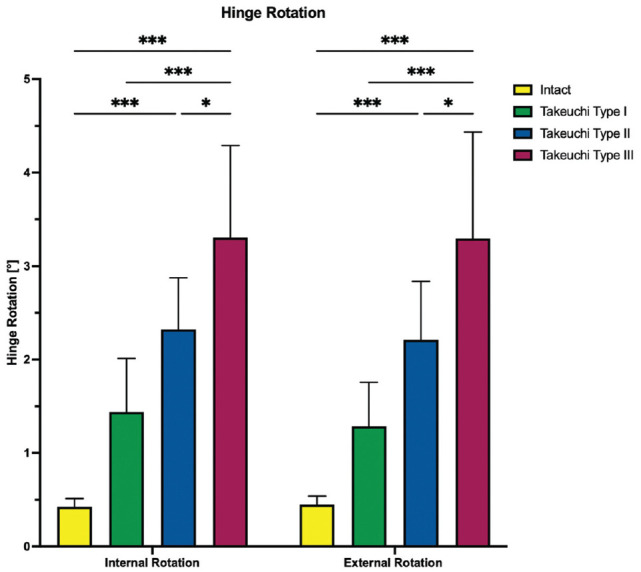
The hinge rotation in internal and external rotation is shown for each testing condition separately. Values are given as mean ± SD. *P < .05; ***P < .001.

**Figure 8.**
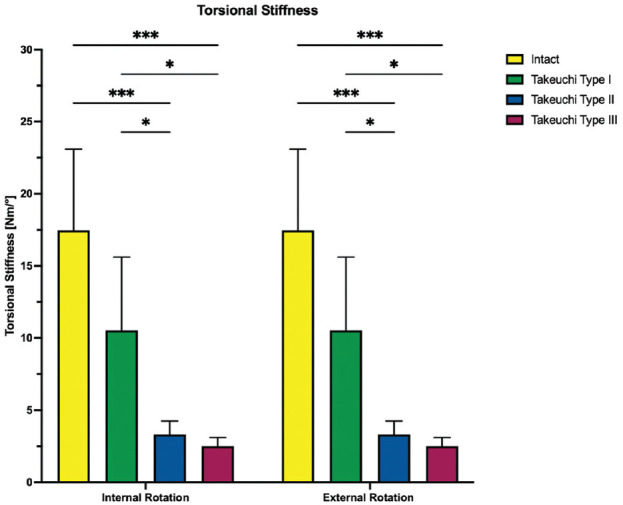
The torsional stiffness in internal and external rotation is shown for each testing condition separately. Values are given as mean ± SD. *P < .05; ***P < .001. ns, not significant.

Regardless of the direction of rotation, Takeuchi type 2 and 3 fractures significantly increased shear displacement and hinge rotation compared with an intact lateral hinge (*P* < .001), resulting in at least 2.2 ± 0.6 mm shear displacement and at least 2.3°± 0.5° hinge rotation for type 2 and 3 fractures in internal and external rotation. With a shear displacement of at least 0.9 ± 0.3 mm and a hinge rotation of at least 1.3°± 0.5° in internal and external rotation, Takeuchi type 1 fractures did not significantly increase torsional instability of the bone-implant construct as compared with an intact lateral hinge (*P* > .112) (Figures 6 and 7). Takeuchi type 2 and 3 fractures significantly reduced torsional construct stiffness compared with constructs with intact lateral hinges, by at least 80% reduction in construct stiffness under rotational loading (*P* < .001). Takeuchi type 1 fractures reduced torsional construct stiffness by at least 39% (*P* < .05) (Figure 8).

## Discussion

The most important finding of the study was that type 2 and 3 LHFs are associated with torsional instability in MOWHTO. Thus, these 2 hinge fracture types might have an increased risk of nonunion as compared with type 1 fractures or intact hinges and might require surgical treatment to restore the biomechanical environment for bone healing.

The integrity of the lateral hinge is crucial for maintaining the biomechanical stability of the MOWHTO.^3,7,31^ In addition to smoking and obesity, LHFs have been discussed as predisposing risk factors to delayed union, nonunion, and loss of correction due to the increased micromovements across the osteotomy gap.^6,18,19,23,28,32^ However, it remains controversial which Takeuchi fracture type should be considered clinically problematic. As the proposed residual stability after LHFs may influence the postoperative rehabilitation protocols and the decision to treat these fractures surgically, Chen et al^3^ recently investigated the Takeuchi fracture type-dependent axial stability of MOWHTO in a synthetic bone model. They showed that Takeuchi type 2 and 3 fractures resulted in a significantly greater medial wedge displacement of 0.5 mm under 800 N of axial loading and a 10% to 26% reduction in ultimate failure load compared with constructs with preserved lateral hinges, whereas Takeuchi type 1 fractures did not affect the axial stability of the bone-implant construct. On the basis of these findings, the authors concluded that type 3 fractures were the most unstable and should be managed cautiously with delayed weightbearing and/or additional fixation. In contrast to these findings, the present study showed that all fracture types caused at least a 28% reduction in axial stiffness with a 0.1-mm increase in axial displacement across the osteotomy, which might be mainly attributed to the different testing protocols (load-to-failure test at 2 mm/s vs nondestructive quasi-static ramp at 720 N in the present study). However, catastrophic failure testing with supraphysiological failure loads of at least 1200 N might not necessarily reflect the clinical problem of nonunion and consequent fatigue-induced hardware failure.

Therefore, in the current study, the Takeuchi fracture type-dependent biomechanical properties of an MOWHTO were analyzed under torsional loading (in addition to axial loading), as shear stress is considered a relevant risk factor for delayed or absent bone formation.^1,2,8^ In comparison to constructs with preserved lateral hinge, type 2 and 3 fractures resulted in at least a 7-fold increase in shear displacement and hinge rotation, as well as an 80% reduction in torsional construct stiffness, whereas type 1 fractures did not significantly affect the torsional instability of the MOWHTO. This may be due to the anatomy underlying the Takeuchi classification. As type 1 fractures extend along the osteotomy plane into the proximal tibiofibular joint, the preserved lateral support provided by the lateral tension band of the proximal tibiofibular joint might stabilize this type of hinge fracture as a biological osteosynthesis, so that these fractures might be considered stable.^22,32^ In contrast, type 2 and 3 fractures extend distally and proximally, respectively, relative to the proximal tibiofibular joint. The lack of stabilization of these fractures by the proximal tibiofibular joint may cause the pronounced torsional instability of the MOWHTO, such that Takeuchi type 2 and 3 fractures should be considered unstable from a biomechanical point of view.^22,32^

The results of the present study are clinically relevant as impaired bone healing of the osteotomy gap, possibly caused by LHFs, remains a major concern in MOWHTO.^20^ In previous in vivo studies of fracture healing, dynamic axial compression was found to promote bone healing, whereas distraction or shear stress inhibited bone formation.^1,2,8^ In particular, a fracture gap >2 mm and a rotational displacement amplitude >0.2 to 1.0 mm have been shown to be critical thresholds for impaired fracture healing.^1,2,8^ Consistent with these findings, Dorofeev et al^4^ reported significantly higher nonunion rates in patients with a dislocated LHF >2 mm (15.4% vs 1.8%). In their retrospective analysis of the procedures of 94 patients, the nonunion rate was particularly increased for LHF with a secondary dislocation >2 mm in the postoperative course compared with undisplaced or primarily dislocated fractures >2 mm during surgery (25.0% vs 6.8% and 1.8%), emphasizing that unstable LHFs lead to impaired bone healing of the osteotomy. Takeuchi type 2 and 3 fractures caused a significant increase in shear displacement >2 mm in both torsional directions, such that these fractures should be considered unstable and might be prone to impaired bone healing. Conversely, Takeuchi type 1 fractures should be considered stable, as these fractures did not significantly affect the torsional stability of the bone-implant construct. Therefore, Takeuchi type 2 and 3 fractures may require additional hinge fixation to reduce the shear stress across the MOWHTO during normal gait to potentially improve bone healing and reduce the risk of nonunions, whereas delayed weightbearing should be considered for type 1 fractures.

### Limitations

The present study has several limitations. First, the biomechanical testing simulated forces acting at time zero when biological factors and osseous integration processes were not considered. Second, cadaveric knee specimens of older age (72.7 ± 7.1 years) were used, which might not necessarily reflect the bone quality of patients treated with MOWHTO. Nonetheless, proximal tibiae of all knees were assessed for BMD to ensure biomechanical testing in nonosteoporotic specimens. Third, an axial load of 720 N and a torsional load of 10 N·m were applied in this study, which might not reflect the loading conditions during normal gait, when the knee bears about 4 times the body weight.^29^ Nonetheless, these loads were chosen to ensure consistency with the current literature and to investigate hinge displacement at predestructive loads to simulate early postoperative rehabilitation.^25^ It should be noted, however, that higher axial loads may have altered the results. Last, the present study only examined displacement at the hinge site under axial compression and torsional loading. Other loading conditions that may also affect bone healing were not investigated.^1,2,8^

## Conclusion

From a biomechanical perspective, Takeuchi type 1 LHFs did not affect the torsional stability of MOWHTO, whereas Takeuchi type 2 and 3 fractures resulted in significantly reduced torsional stiffness, increased shear displacement, and hinge rotation across the osteotomy gap. All Takeuchi fracture types resulted in reduced axial construct stiffness, while axial displacement was not significantly affected by the type of hinge fracture. The observed shear displacement of >2 mm for Takeuchi type 2 and 3 fractures may be indicative of impaired bone healing and may therefore qualify these fractures for hinge fixation to potentially reduce the risk of delayed union and nonunion.
